# Phone messages affect the detection of approaching pedestrians in healthy young and older adults immersed in a virtual community environment

**DOI:** 10.1371/journal.pone.0217062

**Published:** 2019-05-29

**Authors:** Wagner Souza Silva, Bradford McFadyen, Eva Kehayia, Nancy Azevedo, Joyce Fung, Anouk Lamontagne

**Affiliations:** 1 School of Physical & Occupational Therapy, McGill University, Montreal, QC, Canada; 2 Feil and Oberfeld Research Center, Jewish Rehabilitation Hospital (CISSS-Laval), Research site of CRIR, Laval, QC, Canada; 3 Department of Rehabilitation, Université Laval, Quebec City, QC, Canada; 4 Centre Interdisciplinaire de recherche en réadaptation et intégration sociale (CIUSSS-CN), Quebec City, QC, Canada; Tsinghua University, CHINA

## Abstract

**Background:**

Mobile phones are increasingly associated with accidents while walking. Little is known, however, about the impact of phone messaging on the actual perception of other pedestrians. This study aimed to investigate the extent to which the detection of approaching pedestrians is affected by the sensory modality (text or audio) of phone messages in young vs. older adults.

**Methods:**

Eighteen healthy young (24 ± 2.9 years) and 15 older adults (68 ± 4.2 years) performed a phone message deciphering task, an obstacle detection task, and a dual-task condition combining both tasks. Participants were tested while seated and viewing a virtual subway station (VE) in a helmet mounted display. As they were passively moved within the VE one of three virtual pedestrians randomly approached them from the center (0°), right (+40°) or left (+40°). When present, phone message conditions were delivered at obstacle movement onset and presented either as (1) text messages on the screen of a virtual phone or (2) audio messages delivered through earphones. Participants were instructed to press a joystick button as soon as they detected the approaching virtual pedestrian and to report the message content at the end of the trials.

**Results:**

Young and older participants showed delayed obstacle detection times with vs. without text messages. Older adults further showed reduced accuracy of message report for texts compared to audio messages. In both groups, audio messages yielded no difference in obstacle detection time or accuracy of message report compared to the no message condition.

**Conclusions:**

Findings indicate that text messages prolong the detection of approaching pedestrians, suggesting that they compromise safe ambulation in community environments. Older adults, who show larger deteriorations on the obstacle detection and message deciphering tasks, may be at even greater risk of collision. Audio messages could be a safer alternative for on-the-go communication.

## Introduction

The successful avoidance of collisions with surrounding pedestrians is an essential requirement for community ambulation [1]. The ability to circumvent obstacles while walking demands a complex integration of sensory, cognitive and motor resources [2]. Handling simultaneous tasks and distractors while walking further add to those demands and is needed for safe mobility [3]. Moreover, recent evidence indicates that dual-tasking as in texting while walking (TeWW) can be a threat to safe locomotion [4, 5] since it introduces an additional load on perceptual-cognitive resources that are essential for obstacle detection and avoidance [4, 6]. Consequently, pedestrian safety, once mostly associated to street crossing in the presence of vehicles, is now increasingly jeopardized by pedestrian’s habits of dual-task while walking and which lead to compromised ability to avoid collisions with obstacles, including approaching pedestrians [7].

While over 80% of the world population of young adults already own a mobile phone [8], the use among older adults is growing as adaptations in technology are sought to better address their needs [9, 10]. Because coping with phone-related distractors has been shown to compromise the performance of simple tasks such as walking outdoors, the increasing use of mobile technology in community places by young and older individuals is now a major public health issue [11]. Failure to adapt to environmental hazards and difficulties with divided attention would account for the associated risks [12], which can then lead to “inattentional blindness” and reduced gait stability [13]. Receiving phone messages has also been associated with slower gait speed [14], increased trajectory deviation and riskier locomotor behavior in younger adults [8, 15]. In older adults, the potentially dangerous effect of dual-tasking walking could be even higher, mainly due to a reduced ability to negotiate simultaneous attentional demands, leading to a greater increase in dual-task cost or interference [16, 17]. For instance, older adults take longer to start and complete a street crossing task while using mobile phones as compared to young individuals [18]. When prioritizing walking, elderly individuals also show higher cost in text-typing accuracy (increased number of mistakes), while prioritization of texting leads to an increased cost in gait speed (slower gait speed) compared to young individuals [4]. In such a context, it is not clearly understood if the TeWW dual-task costs arise from demands in the allocation of cognitive, motor and/or visual-perceptual resources, all of which are affected by aging [19, 20]. More specifically, the extent to which deciphering phone messages affects the ability of young and older individuals to perceive crucial environmental features such as approaching pedestrians remains to be studied.

Previous research also shows that while both visual and auditory cues can be used to detect and appraise the nature and displacement of an obstacle, obstacle circumvention predominantly relies on vision when the latter is available and accurate [21]. More recently, audio messaging functions were implemented on mobile phones, with the assumption that these would cause smaller interferences with the uptake of visual information. The implementation of such audio technologies allowing hands-free communication is effective in yielding safer driving performance [22]. However, in the context of communication and locomotion, it has not yet been probed whether audio messaging may also be less disruptive than visual text messages and cause less interference with the detection of other pedestrians.

This study used virtual reality (VR) as an ecological, controlled and safe tool to investigate the ability of healthy older versus younger adults to interact with virtual pedestrians (obstacles) approaching from different directions [7]. This study further aimed to determine the extent to which the detection of approaching virtual pedestrians is affected by deciphering phone messages presented in different sensory modalities (visual (text) vs. auditory (voice)). We hypothesized that competition for sensory and attentional resources between obstacle detection and message deciphering will lead to a reduced ability to detect approaching obstacles, which will result in prolonged obstacle detection latency: (1) in the presence versus absence of phone messages; and (2) when messages are displayed as texts, as opposed to audio messages. Additionally, we hypothesized that (3) obstacle detection times as described in (1) and (2) will be compromised to a further extent in older versus younger adults.

## Material and methods

### Study population and setting

Participants included 18 healthy young adults (50% female, aged 24 ± 2.9 years (mean ±1SD)) and 15 older adults (53% female, aged 68 ± 4.2 years). Inclusion criteria were: (1) age between 18 and 30 (young adults) or between 60 and 75 years (older adults): (2) English as the dominant language (as assessed by the short form of the Language Background Questionnaire—LBQ) [23]: (3) right handedness (Edinburg Handiness Inventory) [24]; (4) MoCA score ≥ 26 [25]: (5) regular use of mobile phones (as indicated by pre-screening interview): and (6) normal or corrected-to-normal vision (20/20 on ETDRS chart) and audition (properly repeating audio messages—five correct repetitions out of five messages prior to the beginning of the trial). Exclusion criteria were presence or a history of orthopedic, rheumatologic, or neurological conditions interfering with locomotion and/or upper limb motor control, as well as color blindness. This study was approved by the Ethics Committee of the Center for Interdisciplinary Research in Rehabilitation of Greater Montreal (CRIR). All participants provided written informed consent.

**Fig 1:**
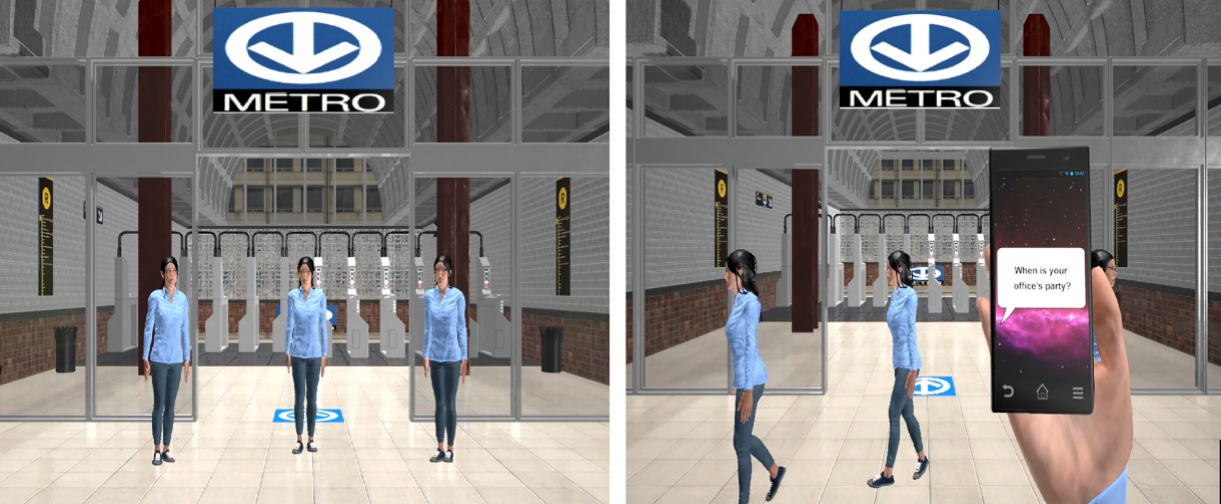
Virtual subway station as seen by the participants through the helmet mounted display. On the left, the picture represents the three virtual humans at the beginning of the trial. The picture on the right represents a condition where a text message was displayed on the screen of a virtual phone during a catch trial (all avatars moving away).

Participants were tested in a single session while seated and viewing a virtual environment (VE) displayed in a helmet mounted display (Nvisor sx60, NVIS, USA) with a 60° diagonal field of view. The VE, controlled with Unreal Engine (Epic Games), simulated a subway station that included a target 11m straight ahead (Fig 1). Three virtual humans were positioned 7m ahead of the participant (straight ahead (0°) and 40° to the right and left from a theoretical point of collision located 3.5 m ahead of the participant). All trials used non-reactive female virtual humans (1.65 m tall) who walked with a neutral gait pattern, as describe previously [21]. The virtual humans were created in Maya LT (Autodesk, U.S.) using pre-recorded motion data collected with a 17-sensor wireless inertial system (YEI technology) in a healthy young participant walking along a straight trajectory at a speed of 1.2 m/s or while turning. Footstep sounds were pre-recorded and timed to the virtual humans’ heel and forefoot contacts with the ground.

A “get ready” sign was displayed before each trial started. The VE then moved automatically creating a backward (posterior-directed) optic flow and a simulated forward locomotor displacement towards the centrally located target at a speed of 1.2 m/s. As the participant reached a forward displacement of 0.5m in the VE, one of the virtual humans randomly approached a theoretical point of collision located 4m ahead on the midline. The other two took one step forward, turned 90° (virtual humans on the right/left) or 180° (virtual human in the center) and immediately walked away in about 0.8s.

Phone messages, when present, were randomly delivered as participants reached 0.5m of forward displacement within the VE, i.e. at the onset of virtual human movement. Seminal work by Gérin-Lajoie and colleagues has shown that the circumvention of moving obstacles while walking occurs in two stages of planning that yield anticipatory estimations (early stage) and final calibration of obstacle clearance (late stage) [1]. Phone messages were delivered at 0.5m to interfere with the early planning phase, i.e, when individuals first detected the approaching obstacle and decided on the strategy to adopt. Messages were controlled for number of words per message, ranging from four to six. Words in each message were controlled for length (letters and syllables), frequency, and comprised of neutral content such as: “I am waiting outside for you” or “I am leaving the gym now”. Message sender was not identified. Text messages were displayed on the screen of a virtual phone that appeared on the right lower quadrant of the view for a duration of 2.3s. Audio messages were played through Bluetooth earphones (SoundPEAT) worn by the participants. For both text and audio messages, a brief ‘beep’ sound preceded the appearance of the message by 0.15s. Throughout the experiment, participants held a joystick (Attack 3 from Logitech) in their right hand with their index finger on a response button. They were instructed to press the joystick button as soon as they detected an approaching virtual human and to refrain from pressing if all virtual humans turned around and walked away. Participants were further instructed to memorize the content of the text message and to report it at the end of each trial. Five trials per direction of obstacle approach (left, center and right) and 5 catch trials (when all virtual humans walked away) were collected under three testing conditions: (1) Single obstacle detection task with no message, (2) obstacle detection and audio message, (3) obstacle detection and text message. Two additional conditions with stationary scenes in which participants simply had to retain the content of audio or text messages were introduced to assess single task message deciphering performance, which was later used for calculation of cognitive dual task-cost. A total of 80 trials were thus collected (5 trials x 2 message modalities (text and audio) x 4 obstacle directions (left, center, right and away) + 20 obstacle-only detection trials (control) + 20 message-only reporting trials (10 for each message modality)).

### Data analysis

Primary outcomes were the accuracy of message report (AMR) and the obstacle detection time (ODT). Secondary outcomes included dual-task costs (DTC), that is the cost induced by the phone message on ODT and the cost of obstacle detection on AMR. In order to determine ODT, the time difference between the onset of obstacle displacement and the moment when participants clicked the joystick button was calculated. AMR was calculated as the percentage of messages that were fully reported by the participant (i.e. all words included in the message) out of the total number of messages received in a given condition. DTCs were calculated as the percentage change in performance from the single task to the dual task condition, such that DTC = [100 X (Dual-Single)/Single]. Data analyses were done in MATLAB v.9.3 (MathWorks, U.S).

### Statistical analysis

The effects of message modality and direction of obstacle approach (within-subject factors), as well as the effect of age group (between-subject factor) on the different outcomes listed above were examined using repeated measure mixed models. The mixed model approach accounts for possible large between-subject heterogeneity and is also tolerant to small and unequal sample sizes [26]. Combined covariance structures and a random coefficient structure were used. Compound symmetry was further ascertained by evaluating the fit of the data and deviations from model assumptions using residuals’ analysis. For AMR, the model included 2 groups x 2 message modalities x 3 obstacle directions whereas for ODT the model was based on 2 groups x 3 message modalities (including the no-message conditions) x 3 directions of obstacle approach. Post-hoc analyses were conducted as applicable using pre-planned pairwise comparisons. The sample size was determined to allow effect sizes between 0.3–0.4 across all outcomes, confirmed a posteriori. The probability level was set at p < 0.05. Analyses were done in SAS v.9.4.

## Results

Young adults reported 17 ± 2.6 years of education and 8.4 ± 1.1 years of experience with mobile phones with a daily use of 3.1 ± 1.3 hours. Thirty percent of participants reported having a history of accidents while using their mobile phone. Older adults reported 16 ± 3.4 years of education and 7.8 ± 2.7 years of experience with mobile phones with a daily use of 1.6 ± 1.2 hours. None had a history of accidents while using their mobile phone.

### Effects of message modality

**Fig 2:**
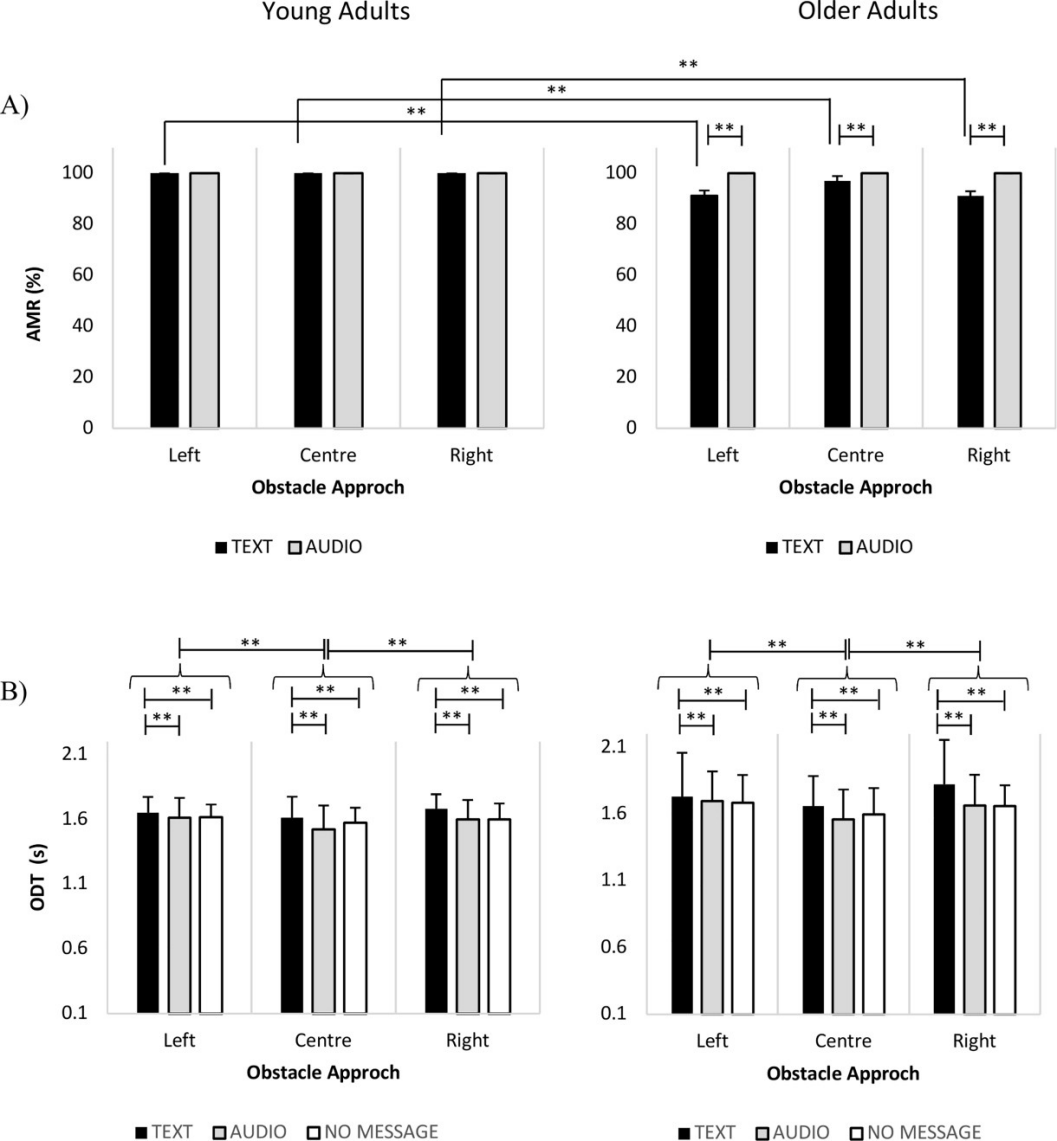
(A) Mean values (± 1SD) of accuracy of message reports (AMR) for phone messages delivered as text and audio messages for young (left) and older (right) adults. (B) Mean values (± 1SD) for obstacle detection times (ODT) for text and audio messages, as well as the no-message condition in the two ages groups. Statistically significant results are indicated. Symbol indicates **p < 0.01.

For the single task message deciphering condition (without obstacle detection), both groups obtained 100% in AMR. The analysis of how participants dealt with the different sensory modalities of phone messages (Fig 2) revealed a significant interaction of group X sensory modality for AMR (p < 0.001) and a main effect of sensory modality for ODT (p < 0.001). Post-hoc analyses showed that AMR was reduced in older adults compared to young adults when exposed to text (p < 0.001) but not audio messages (p = 1.00) for all directions of obstacle approach. Text messages also lead to longer ODT compared to the audio and no-message conditions (p < 0.001) for all obstacle directions in both young and older adults. No significant differences in ODT were observed between the audio message modality and the no-message condition (p = 0.4). However, there was a main effect due to obstacle direction, with increased ODT for both left and right obstacle vs. the center (p < 0.001) obstacle across all message modalities.

### Dual-task costs

The DTC induced by the obstacle detection task on the AMR and the cost of attending to phone messages on ODT were calculated and are reported in Table 1. The analysis of DTC for AMR first revealed no cost (DTC = 0) when messages were delivered in the audio modality both for young and older adults. For text messages, however, significant interaction effects of group X message modality (p < 0.001) were observed, with greater DTCs on AMR found in older adults when deciphering text messages (p < 0.001) compared to young individuals for all directions of obstacle approach.

As for the cost of deciphering phone messages on ODT, no significant differences were observed between groups (p = 0.4) and no significant cost was associated with deciphering audio messages (p = 0.5). Significant interaction effects between message modality and obstacle direction (p < 0.001, however, showed greater cost imposed by text messages compared to audio messages in the presence of obstacles approaching from the center (p < 0.001) and right (p < 0.001) directions.

**Table 1:** 

**Cost of obstacle detection on accuracy of phone message (text and audio) report**			
**DTC to ARM**			
**Phone message modality**	**Obstacle**	**DTC in %**	**DTC in %**
	**approach**	**Young adults**	**Older adults**
	Left	0.0	8.7 ± 16*
Text messages	Center	0.0	3.0 ± 8.0*
	Right	0.0	8.9 ± 14*
	Left	0.0	0.0
Audio messages	Center	0.0	0.0
	Right	0.0	0.0
**Cost of deciphering phone messages (text and audio) on obstacle detection time**			
**DTC to ODT**			
**Phone message modality**	**Obstacle**	**DTC in %**	**DTC in %**
	**approach**	**Young adults**	**Older adults**
	Left	1.2 ± 5.6	2.2 ± 10
Text messages	Center	1.9 ± 8.0*	4.1 ± 8.9*
	Right	4.8 ± 8.5*	9.7 ± 16.2*
	Left	-1.3 ± 7.2	1.0 ± 7.5
Audio messages	Center	-3.9 ± 8.2	-2.1 ± 8.7
	Right	-0.6 ± 6.4	0.1 ± 8.8

Dual task costs (DTC) for accuracy of message response (AMR) and obstacle detection time (ODT) in the young and older adults. Dual-task cost (DTC) incurred by the detection of obstacles on the accuracy of message reports (AMR) and phone messages deciphering on the obstacle detection time (ODT). DTC represented as positive and negative values indicate, respectively, an increased and a reduced cost compared to the single task conditions. Symbols indicate *p < 0.01, **p < 0.01 compared to left approach.

## Discussion

Previous studies have addressed obstacle avoidance in the context of walking. Those studies, however, did not address the ability to visually detect an approaching obstacle or the extent to which such ability is compromised by distractors presented under different sensory modalities in the context of pedestrian approach. To our knowledge, this is the first study to explore the aforementioned aspects in both young and older adults devoid of walking demands. This study has some limitations which include the use of a VR-based experimental setting and the interaction with non-reactive female virtual humans as the only version of approaching pedestrians. Previous evidence, however, indicate that VR is a reliable alternative towards the study of human perception, including sense of presence and consciousness[27]. In fact, even when perception was paired with locomotor strategies in VR, responses have been shown equivalent to those observed in the real world [28]. Furthermore, the use of only female pedestrians may also not represent the variability of contexts within public spaces of the real world, and hence bring limitations to the generalizability of our results. However, this single-sex obstacle design allowed for better consistency across trials given the fact that the physical characteristics of the interferer, including sex, may influence interactions in virtual environments [29]. Collectively, results show that deciphering phone messages can compromise pedestrian detection, especially when the message is delivered as text. Results further show that older adults exhibit increased dual-task interference when simultaneously deciphering text messages and detecting approaching obstacles.

### The sensory modality of phone messages differentially affects obstacle detection

Results showed that text messages caused delayed ODTs compared to the audio and no-message conditions, both in young and older adults, indicating that the modality of phone messages matters. Previous evidence suggests that when two objects of interest are presented in close temporal proximity among distractors, the identification of the first object evokes a perceptual deficit for perceiving the second [30]. This perceptual deficit would serve the purpose of supporting the conscious retention of the first object identity for subsequent report. In contrast, if the two objects of interest are presented with a longer time lag between them, no perceptual deficit is observed. A similar process of interference for stimuli presented in close temporal proximity may explain why participants of the present study showed longer ODTs when exposed to text messages delivered simultaneously to the interfering movement onset. The fact that audio messages left ODTs unaltered compared to the no-message condition, however, implies less perceptuo-cognitive interference when the two stimuli are presented under different (audio-visual) as compared to shared (visual-visual) sensory resources. This could suggest the presence of separate attentional resources for the visual and auditory sensory modalities [31]. The practical implication of this finding is that audio messages could represent a safer alternative to text messages for pedestrian interaction and collision prevention, assuming that what is observed for perception applies to action, i.e. the actual implementation of avoidance strategies during locomotion.

### Different dual-task costs in older vs. young adults

Our results also showed that while both young and older adults took longer to detect the approaching obstacle when exposed to text messages, only the older adult group showed a reduced ability to decipher the text content. The presence of dual-task interference where both tasks of interest, cognitive and motor, are compromised in older adults is not surprising and has commonly been observed in the literature, including studies on TeWW [4, 32]. In the context of this study, this dual-task cost may be attributed to the decline observed in a variety of visual-perceptual functions [33, 34], memory [35], executive functioning [36], motor function [36] and sensorimotor integration [20] observed with older age. Additionally, while both young and older adults had a similar number of years of cell phone experience, older adults reported half of the hours dedicated daily to interact with their phones as compared to young individuals. Given that improved allocation of attentional control and reduction of dual-task cost have been associated to training [37], a shorter practice in handling cell-phone tasks on a daily basis may have contributed to the compromised performance seen in older adults. The observation that younger adults preserved their ability to decipher messages while concurrently performing another task also aligns with previous literature on TeWW which shows that younger adults seemingly text during split-belt walking [38] and prioritize texting over walking during locomotion in indoor and outdoor real environments [4]. This preserved ability to decipher phone messages, however, may mislead young adults into misjudging the risks involved with using their mobile phone while walking. As for older adults who represent a substantial and growing number of mobile phone users, current data suggest that they could be at even greater risks of collisions and injuries due to further delays in detecting approaching pedestrians.

### Effect of obstacle direction

Results showed that central approaching obstacles led to faster ODT across all tested conditions compared to diagonal approaches (left and right obstacles). Previous findings from our laboratory, employing a similar obstacle avoidance paradigm during locomotion, showed that prior to the onset of obstacle displacement, the head and gaze are aligned with the end destination [39]. We hypothesized that this alignment of head and gaze with final destination which was represented by a centrally located blue target may have facilitated and assisted in preserving the ability to detect a centrally approaching obstacle, despite the occurrence of text messages. This robust detection of a centrally approaching obstacle is actually advantageous for locomotion on foot, given that such an obstacle can only be avoided through a change in walking trajectory, which demands an initiation of avoidance strategy at a further distance from the obstacle compared to diagonal approaches [21]. Furthermore, due to an unbalanced speed of visuospatial processing associated with a lager parieto-frontal network in the right cerebral hemisphere compared to the left [40], visuospatial attention in right handed healthy individuals tends to be lateralized to the right hemisphere, which could result in more efficient detection of motion happening in the left hemifield depending on the context of cueing visual fixations [41]. As a result, obstacle detection may be facilitated and preserved when approaching from the left side of space, but not from the right space. This could explain the smaller DTC seen in the presence of left approaching obstacles compared to center and right approaches as observed in our study. Text messages, when present, were also introduced from the lower right corner of the virtual scene. That may have caused further visual interference to the perception of the right approaching obstacle placed on the same visual hemifield from where text messages originated. However, because in daily life right handed individuals hold their phones with their right hand, often bringing their devices up through their right visual hemifield, the design of this study was kept as such for better generalizability of results to real-life situations.

## Conclusions

Our findings suggest that text messages do pose a threat to the safety of individuals interacting in a community environment due to interferences caused by the pairing of text deciphering to the visual detection of approaching pedestrians. This dual-task interference depends on the sensory modality of the phone message and can vary as a function of the direction of obstacle approach. The pattern of dual-task interference also depends on age, as young adults exposed to text messages prioritize the message deciphering task over obstacle detection, while older adults show a concurrent deterioration in their message deciphering and obstacle detection abilities. While it seems intuitive that audio would be less disruptive than text messages, evidence that dual-task interference is present when a secondary task is not using the same sensory modality of the primary task [42, 43] led us to expect some extent of cost in response to audio messages compared to when no messages were present, which did not occur even among older adults. Finally, this study shows that audio messages stand out as a safer alternative to text messages, allowing for better retention of message content in addition to more efficient perception of approaching obstacles. Future research should target how the perceptual behaviors observed here translate into locomotor contexts such as TeWW in urban areas.
